# Effects of Awakening and the Use of Topical Dexamethasone and Levofloxacin on the Cytokine Levels in Tears Following Corneal Transplantation

**DOI:** 10.1155/2014/570685

**Published:** 2014-10-12

**Authors:** Mariann Fodor, Goran Petrovski, Dorottya Pásztor, Péter Gogolák, Éva Rajnavölgyi, András Berta

**Affiliations:** ^1^Department of Ophthalmology, University of Debrecen Clinical Centre, Nagyerdei Krt. 98, Debrecen 4012, Hungary; ^2^Department of Ophthalmology, Faculty of Medicine, University of Szeged, Korányi fasor 10-11, Szeged 6720, Hungary; ^3^Stem Cells and Eye Research Laboratory, Department of Biochemistry and Molecular Biology, Faculty of Medicine, University of Debrecen, Nagyerdei Krt. 98, Debrecen 4012, Hungary; ^4^Department of Immunology, University of Debrecen Clinical Centre, Nagyerdei Krt. 98, Debrecen 4012, Hungary

## Abstract

*Objectives*. To study the short-term effect of eye opening and use of topical dexamethasone phosphate 0.1% and levofloxacin 0.5% on the cytokine levels in human tears.* Methods*. Prospective experimental design was used for tear collection from eyes of 10 healthy controls and 20 patients four days after penetrating keratoplasty (PKP) at awakening and after instilling dexamethasone or levofloxacin. The concentrations of different cytokines were measured by cytometric bead array.* Results*. At eye opening, IL-6 levels were higher in the PKP group as compared to the controls. Thirty minutes later, the released levels of IL-10, IL-13, IL-17, IFN*γ*, and CCL5 increased in controls, while CXCL8 decreased in both control and PKP groups. The release of the cytokines remained stable after 30 mins except for IFN*γ*, which showed a decrease in the controls following levofloxacin instillation. No short-term effects of the topically used dexamethasone and levofloxacin could be detected on the cytokine levels in controls and after PKP.* Conclusions*. Evidence of changes in the levels and time course of tear cytokines after awakening or eye opening could be established and the short-term confounding effects of dexamethasone and levofloxacin on the levels of released cytokines in human tears could be excluded.

## 1. Introduction

The preocular tear film is essential for maintaining ocular surface homeostasis and the tear samples may be a useful source of information for understanding the molecular mechanisms behind several eye diseases. With improvement of analytical methods and high-throughput technologies, studies quantifying the levels of mediators in human tears have gained increased value [1–4], while studies performed during the postoperative period are desired.

Beside accurate sampling of tears, many confounding factors can modify or affect the results obtained from tear-release studies: timing of tears' collection (open- or closed-eye phase), instillation of eye drops, age, sex, tear flow rate, collection technique, different methods for measuring cytokines, pooling, centrifugation, and storage of the samples [1–15].

The tear film has been extensively studied in the open-eye state, and some studies have shown that eye closure results in a profound change in tear composition [1, 14, 16, 17]. During sleep, subclinical inflammatory state takes place, which has a great importance in the pathomechanism of several eye diseases [1, 16]. The closed-eye state induces the recruitment and activation of polymorphonuclear (PMN) cells [17], and because of reduced tear film clearance, the cytokine levels may be modified as well. The levels of IL-1, IL-6, IL-8, IL-10, IL-12p70, and TNF-*α* have been investigated previously following three-hour eye closure, without providing information about the time needed for tear film recovery from the closed-eye state [3, 18]. Information about recovery time is important for valid comparative studies of tear cytokines. Closed-eye tear samples, which have been recovered immediately after overnight eye closure, have been first described by Sack et al. [16]. Changes in the levels of cytokines, proinflammatory mediators, and growth factors during overnight eye closure, however, have been of limited research interest so far [17].

The timing of tear sample collection is not a standardized procedure, although the time after awakening and after application of local treatment can influence the levels of several mediators, thereby modifying the overall clinical situation. Topical application of fluoroquinolones and steroids has become a common treatment after ophthalmic surgeries including corneal transplantation (keratoplasty) [19, 20], but very little is known about their short-term effects on the cytokine levels of the tear film.

To study the short-term effects of awakening and the use of topical dexamethasone and levofloxacin on cytokine levels in human tears, we measured the concentration of multiple cytokines in tear samples of controls and patients treated by penetrating keratoplasty (PKP). Additional evidence on the alteration of a wide range of tear cytokines collected after awakening or eye opening could be demonstrated, while short-term confounding effects of dexamethasone and levofloxacin on the cytokines in human tears could be excluded.

## 2. Patients and Methods

### 2.1. Subjects, Tear Collection, and Analysis

All human tear samples were obtained with a written patient consent according to the tenets of the Declaration of Helsinki and sample collection procedures were approved by the Regional Ethics Committee of the University of Debrecen.

Within a prospective study, nonstimulated tear samples were collected from 20 eyes of 20 patients (mean age: 66.7 years, SD: 16.9) four days after PKP and from 10 eyes of 10 healthy controls (mean age: 68.6 years, SD: 16.9). All donor corneas were preserved in Optisol-GS (Bausch & Lomb, USA) for at most 7 days. Routine medications, local corticosteroids (dexamethasone phosphate 0.1% (Maxidex, Alcon)) and antibiotics (levofloxacin 0.5% (Oftaquix, Santen Oy)), were applied at four time points (four eye drops of each drug per day) after corneal transplantation. None of the patients received systemic anti-inflammatory therapy (intravenous or oral corticosteroids). Exclusion criteria for controls included active inflammatory or infective systemic or ocular diseases, current treatment with systemic or local anti-inflammatory drugs, and patients with contact lenses. Eyes with previous ocular surgery or trauma were also excluded from the study. The causes for corneal transplantation were keratoconus, bullous keratopathy, transplant rejection, corneal vascular leucoma, and corneal scar.

Before every tear collection, the anterior ocular status of each subject was carefully assessed; a slit-lamp examination under low illumination was performed to avoid reflex tearing. Sample collection was always performed by the same person. All the study participants were asked not to open their eyes before sample collection. At day 4 after surgery, the reepithelisation of the transplanted cornea in our study reached in all cases a continuous epithelial layer. Tears were collected from each eye at 6:00 (upon eye opening after overnight sleep), 6:30, 6:40 (10 mins after instilling an eye drop of dexamethasone), 6:50, and 7:00 a.m. The eyes were treated with one eye drop of dexamethasone phosphate at 6:30 a.m., followed by one drop of levofloxacin at 6:50 a.m. All drops were instilled into the inferior* cul-de-sac*. Drops were administrated by a doctor to ensure strict compliance with the administration regimen.

Nontraumatic tear collection was carried out with capillary tubes from the inferior meniscus without topical anesthesia for 2 mins and then the total volume of each collected tear sample was registered. The samples were immediately transferred to Eppendorf tubes and frozen at –80°C without centrifugation within 15 min from collection. To avoid pipetting and dilution errors, collected tear samples of <4 *μ*L were excluded. Occasionally, tear collection could not be carried out due to dry eyes.

The concentrations of IL-1*β*, IL-6, IL-10, IL-13, IL-17, IFN*γ*, IL-8/CXCL8, IP-10/CXCL10, and RANTES/CCL5 were measured by the cytometric bead array (CBA) method. Combined FlowCytomix Simplex Kits were used with the appropriate FlowCytomix Basic Kit with minor modifications from the manufacturer's instructions (eBioscience, Bender MedSystems GmbH, Vienna, Austria). Briefly, 15 *μ*L of tear samples (in some cases, in Assay Buffer 2–10x diluted samples) or serial dilutions of mixed standard cytokines were added to 15 *μ*L suspension of fluorescent cytokine capture beads on multiwell filter microplates. 15 *μ*L of biotin conjugated anti-cytokine antibody was added to the wells, and the plates were then incubated for 2 hours on a microplate shaker. The wells were emptied and washed with a vacuum filtration manifold. Phycoerythrin-conjugated streptavidin was added to the wells followed by additional incubation for 1 hour and washing as described above. 150 *μ*L assay buffer was applied to the wells; then multiparametric data acquisition was performed on a FACS Array cytometer (BD Biosciences Immunocytometry Systems, San Jose, CA). Data were analyzed with the FlowCytomix Pro 2.3 software. Additional serial dilutions of the standard were applied to obtain better sensitivity and, therefore, modified standard curves were generated in the analysis. In cases of diluted samples, the end concentration of the samples were calculated accordingly. The detection limits were IL-1*β*: 4.2 pg/mL; IL-6: 1.2 pg/mL; IL-10: 1.9 pg/mL; IL-13: 4.5 pg/mL; IL-17: 2.5 pg/mL; IFN*γ*: 1.6 pg/mL; IL-8/CXCL8: 0.5 pg/mL; IP-10/CXCL10: 6.0 pg/mL; RANTES/CCL5: 25 pg/mL.

### 2.2. Statistical Methods

The quantities of mediators released into tears were calculated as products of concentrations (pg/*μ*L) and tear volumes (*μ*L) collected over 2 mins. Continuous variables were described in each patient group and in the overall study sample using standard statistics.

Analysis of correlation between clinical data and CBA data was performed using unadjusted as well as adjusted estimation procedures. Cytometry data were geometrically averaged across measurement occasions to derive a single value for each patient. Spearman's correlation coefficients were used to estimate unadjusted correlations. Adjusted estimates were semipartial correlation coefficients derived from multiple linear regression models with patient group, age, sex, and total volume of collected tear fluid as explanatory variables and each cytometry variable in turn as the outcome variable.

Unadjusted intergroup differences at baseline (6:00 a.m.) and at 6:30 a.m. were tested using the* t*-test or a nonparametric equivalent subject to distributional assumptions being satisfied. Age-adjusted comparisons were carried out using multiple linear regression.

Within-group differences of release levels across measurement occasions and between-group differences of release levels adjusted for the previous measurement were assessed using multiple linear regression with adjustment for age and with an interaction term between patient group and measurement occasion. The models were checked by testing for heteroskedasticity and regression specification errors.

Variables were transformed to improve normality unless this failed to produce substantially better fit assessed by normality of standardized residuals.

The statistical package applied was Stata version 11.2. The significance criterion was set at *α* = 0.05. All *P* values shown are from adjusted analyses unless stated otherwise. Power analysis was performed to justify the number of patients enrolled in the study.

## 3. Results

Differences in gender, age, and volume of collected tear fluids could not be detected between the patient and the control group (Table 1). The average collected tear fluid volume was 10.1 *μ*L (SD: 5.9) in PKP group and 8.8 *μ*L (SD: 9.4) in control group. The variable amounts of collected tear fluid were calculated and the release levels of the mediators were measured.

The correlation analyses between clinical data and cytometry bead array data using unadjusted as well as adjusted estimation procedures are shown in Table 2. Age with CXCL8 and total tear volume with IL-13 correlated significantly, irrespective of the estimation procedures. Age with IFN*γ* and IL-10 and total tear volume with CXCL8 correlated significantly if adjusted estimation procedures were used and total tear volume with IP-10 correlated significantly if unadjusted estimation procedure was used.

**Figure 1:**
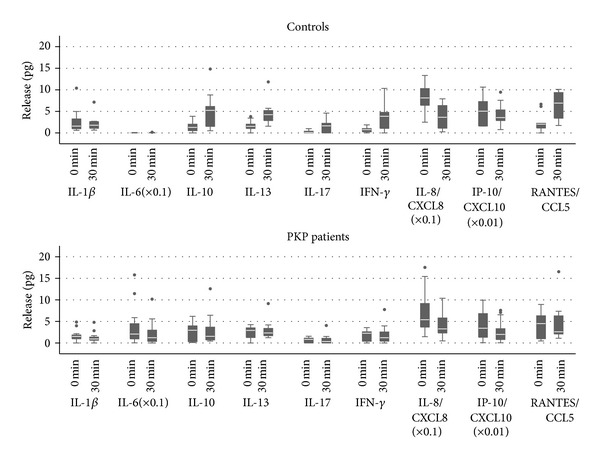
Concentration of mediators in released tear samples at baseline (0 min, awakening or eye opening) and 30 minutes after eye opening in PKP patients and controls. Values shown were obtained by applying scaling factors where indicated (∗).

**Figure 2:**
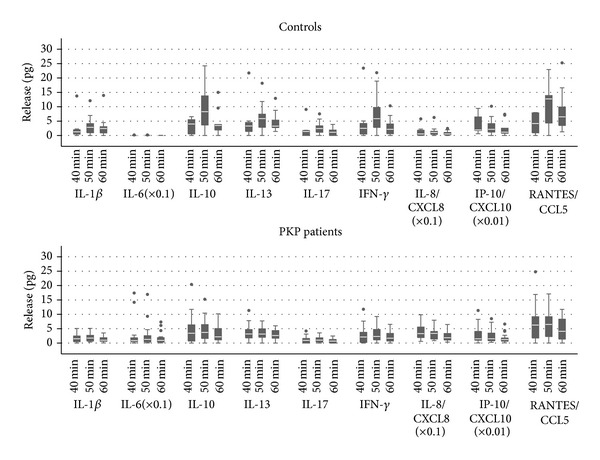
Concentration of released mediators 40-50-60 minutes after eye opening in PKP patients and controls. Treatment with one eye drop of dexamethasone phosphate at 30 minutes and one drop of levofloxacin at 50 minutes after awakening or eye opening was carried out. Values shown were obtained by applying scaling factors where indicated (∗).

At baseline (6:00 a.m., awakening or eye opening), higher IL-6 level was detected after PKP as compared to controls (*P* < 0.0001 unadjusted, *P* = 0.0153 adjusted for age). Thirty minutes after eye opening the release of IL-10 (*P* = 0.004), IL-13 (*P* = 0.0005), IL-17 (*P* = 0.007), IFN*γ* (*P* = 0.003), and CCL5 (*P* = 0.0009) increased in the controls, while the release of CXCL8 decreased (*P* = 0.0007). Under the same conditions, the release of CXCL8 decreased in the PKP group as well (*P* = 0.02) (Figure 1 and Table 3). The cytokine release remained stable after 30 mins after eye opening and throughout the time interval of examination except for IFN*γ* which decreased after using levofloxacin eye drop in the control group (*P* = 0.02) (Figure 2).

## 4. Discussion

The question of the effects of eye closure on tear film composition was first raised 35 years ago [21], and subsequent investigations on the effects of overnight eye closure as a contributing factor have been reported to explain the discrepancies in data reported in the literature [1, 14, 16, 17]. Indeed, tear collection time, flow rate, stimulus conditions, and collection techniques [14, 22] may contribute to the variation of cytokine concentrations measured in tear samples and may influence the protein profile of the tear samples [5–7, 9]. Besides the precise and reproducible determination of tear protein levels, the uniform interpretation (concentration versus release), the time after opening the eye, and the local treatment applied have crucial impact on the levels of different mediators. Without understanding how various soluble factors influence the released and measured cytokine levels, the integration and reliability of the data obtained from different studies remain problematic. In this study changes in cytokine levels measured in tear fluids of healthy controls and PKP patients in response to awakening or eye opening and treatment with topical dexamethasone phosphate and levofloxacin were studied. The effects of awakening on the release of nine cytokines in human tear samples were investigated. We found that the levels of IL-10, IL-13, IL-17, IFN*γ*, and CCL5 were lower in the closed-eye tears, while the level of CXCL8 was higher at eye opening when compared to the values measured 30 mins later. These findings are in line with numerous studies showing alterations in tear fluid composition after awakening [1, 3, 10, 14, 16, 17]. During sleep or eye closure, physiological changes occur in the ocular surface causing a local subclinical inflammatory state [1, 16]. Studies have shown that eye closure results in a profound change in the composition, origins, turnover, and physiological functions of the tear film with the accumulation of various cytokines and mediators [17]. A specific diurnal alteration pattern of IL-1*β*, IL-6, CXCL8, IL-10, IL-12p70, and TNF*α* has been found showing the importance of the timing of tear collection during the day [2]. It is noteworthy that the examination of different cytokines present in overnight tears has been reported mainly in control [1, 10] and allergic eyes [14].

In the present study, only the IL-6 level was higher in the tear samples of patients after PKP at awakening compared to the controls; this was in accordance with our earlier reports [23]. IL-6 is a highly sensitive indicator of various types of irritative eye diseases and high levels of it have been detected in closed-eye tears [10]. In contrast to these results, we demonstrated that awakening caused no significant alteration in IL-6 levels in both the controls and the patients. Previously, Thakur et al. could not detect IL-6 in normal open-eye tear samples that could have been due to inappropriate sensitivity of the IL-6 assay used for detection [10]. The level of the anti-inflammatory cytokine IL-10 increased after awakening, which is consistent with previous reports showing the induction of a subclinical inflammatory state during 3-hour eye closure [3].

Among the TH2-related cytokines, IL-13 is known as a normal constituent of human tears [15], although it has also been detected in pathological tear samples [24]. In our study, the release level of IL-13 increased after eye opening in the controls, but no alteration was observed after awakening in the patient group. Low levels of IL-13 have been detected in severe keratoconus [25], as well as high levels in Graves' orbitopathy [26]. The exact function of IL-13 upon eye closure requires further investigation.

Interestingly, 30 mins after eye opening the release level of CXCL8 decreased in our patient group, while the concentration of the other cytokines remained unchanged, pointing to the fact that a four-day use of topical dexamethasone and levofloxacin can profoundly modulate the level of the measured cytokines. Furthermore, these treatments can also reduce cytokine levels due to awakening. A higher level of CXCL8 was found in the closed-eye tears of the controls, consistent with some previous reports showing that CXCL8, acting predominantly as a chemoattractant, was increased in the tear fluid during sleep [10, 12, 17].

Previously, Jun et al. [27] have shown that lower levels of CCL5 were found in keratoconic patients but contact lens wear had no significant effect on it in our study and a significantly increased CCL5 level was detected after awakening in the controls showing that this chemokine may have less function during sleep.

Recently, the roles of IL-17 and IFN*γ* have been investigated in ocular diseases; however, to the best of our knowledge, the effect of awakening on these cytokines has not yet been investigated. The tear levels of IL-17 and IFN*γ* were higher 30 mins after awakening when compared to those immediately after eye opening.

The short-term effect of topical treatment on the level of cytokines released in tears has previously been studied without involving sample collection at 24 hours [14]. Another study in which exclusion criteria for patients treated by anti-inflammatory agents has also been published [12]; however, there are many patients who cannot be left without medication for such a long period of time. To the best of our knowledge, this is the first report demonstrating the lack of short-term effects of topical dexamethasone and levofloxacin on the cytokine levels in human tears even after PKP, since these treatments did not influence the release of the examined cytokines within 30 minutes from administration (except IFN*γ*).

Steroids have been widely used in ophthalmology [28] and they exert a strong anti-inflammatory effect on inflammatory cytokine production. The mainstream in the prophylaxis of immune reactions after PKP is still the administration of topical corticosteroids during the postoperative period, but the short-term effects of steroids on cytokine levels in the human tear film have not been studied so far. Our results demonstrate that one drop of dexamethasone had no short-term effect on the cytokine levels in tears, although long-term effects were not monitored after PKP, when the patients received topical steroids continuously. Regulation of cytokine levels in the human tears and the duration of their downregulation await further analysis. Topically applied dexamethasone did not cause significant changes in colony counts on a healthy conjunctiva [20] and the expression of several proinflammatory genes has been shown to be attenuated by dexamethasone [29], and this treatment* in vitro* has been found to inhibit matrix protein deposition and fibrosis, thereby maintaining corneal clarity [29]. Steroids retard corneal epithelial healing [30] and systemic administration of betamethasone was able to suppress the inflammatory cytokine expression (IL-1, IL-6, and IL-8) in a rat cornea model [31].

Fluoroquinolones are antibacterial agents with bactericidal activity. Levofloxacin, like other quinolones, has an immunomodulatory action on the cytokine production at an early transcriptional step of cytokine gene expression [32, 33]. Suppression of IL-6 by quinolones has been proven* in vitro* [34]. Fluoroquinolones penetrate efficiently into eyes with disrupted epithelium [35]. After a short-term usage of levofloxacin eye drops, high corneal and aqueous humor concentrations of this quinolone can be reached [19, 36, 37]. Four days after PKP the epithelium is not yet perfectly intact, so high aqueous humor concentration of levofloxacin is presumed; however, no information is available on the short-term effects of the use of topical levofloxacin on the cytokine levels in human tears. Our study followed the changes in the cytokine levels after 10 mins from instillation of levofloxacin eye drops. As 15 mins is usually needed for the penetration of levofloxacin to the cornea, it may persist in the tear film causing an alteration in the level of cytokines [19, 36, 37]. When a drop of one medication is followed closely by drops of other medications, substantial washout and pH change may occur [36]. We set a 10 min interval between the administrations of each eye drop to minimize the above effects. Administration of levofloxacin has been found to modify the levels of IL-1, IL-6, IL-8, and TNF-*α* in a time- and/or dose-dependent manner [38, 39]. In our study, after administration of a levofloxacin eye drop the release of the cytokines remained stable for 10 minutes except for IFN*γ*, which decreased in the controls. We could not detect any short-term effects of topically used levofloxacin on the cytokine levels in tears after PKP. A possible explanation for this effect could be that levofloxacin is one of the fluoroquinolones, which causes less influence on the concentration of released cytokines as compared to other drugs. In addition, the 10 mins time may have been enough for the eye drop to wash out, but not enough to cause a significant effect.

The large plethora of mediators found in the tears makes it impossible to conclude from the present study that levofloxacin and dexamethasone have no short-term effects on the levels of all mediators found in tears. This study was restricted to a total of 9 measured cytokines, which can be further expanded with the availability of high-throughput multiplex bead array technology. Nevertheless, it is important to emphasize that our results highlight the fact that the levels of many cytokines and mediators in the tear fluid change after awakening, and levofloxacin and dexamethasone have little short-term effect on these mediators. Our findings raise several questions as to whether there are other cytokines and chemokines that could be affected after short-term use of steroids or antibiotics, and the degree in which other topical treatments may affect the level of the tear mediators. Since steroid eye drops are often administered in ocular surface disorders, it is necessary to investigate the long-term effects of prolonged use of corticosteroids on tear mediators.

To the best of our knowledge, this study presents for the first time the fact that not only normal, but also pathological, conditions may exert limited and/or short-term effects after the use of levofloxacin and dexamethasone on the levels of tear cytokines. Our data demonstrate that the release of IL-1*β*, IL-6, and CXCL10 is not altered after successive administration of levofloxacin and dexamethasone in both the control and the pathological tear samples. Since cytokines and other mediators have been implicated in the pathophysiology of a wide range of corneal diseases, gaining knowledge about the different confounding factors affecting the cytokine levels in tears was shown to be appropriate. We further demonstrated the importance of timing and the urgent need of commonly accepted guidelines or collection techniques for tear collection. Collection of tear samples between 9 and 12 a.m. [24] and the arguments suggesting morning collection before the first eye drops are instilled [40] need better coordination and/or revision. In summary, additional evidence for the concentration changes in a wide range of tear cytokines after awakening or eye opening could be established and the short-term confounding effects of dexamethasone and levofloxacin on the cytokines present in human tears could be excluded.

## Figures and Tables

**Table 1:** Clinical data and total volume of tears collected by each subject in PKP patients and controls.

	PKP patients	Controls	Statistics
Gender (female : male)	12 : 8	7 : 3	Fisher's exact test: *P* = 0.70
Age (mean; (SD))	68.7 (16.9)	66.7 (16.9)	Wilcoxon rank-sum test: *P* = 0.83
Collected total tear volume by each subjects (*μ*L, mean; (SD))	50.2 (22.9)	42.1 (39.7)	Wilcoxon rank-sum test: *P* = 0.07

**Table 2:** Correlation analysis between clinical and cytometry bead array data (geometric mean of the released cytokines) using unadjusted as well as adjusted estimation procedures (significant differences are shown in bold).

	IFN*γ*	IL-17	IL-10	CXCL8	IL-6	IL-13	IP-10	IL-1	RANTES
Age									
Unadjusted correlation	−0.27 (*P* = 0.14)	−0.36 (*P* = 0.05)	−0.30 (*P* = 0.11)	**0.64 (** **P** < 0.001**)**	0.26 (*P* = 0.16)	−0.003 (*P* = 0.99)	0.30 (*P* = 0.11)	−0.24 (*P* = 0.20)	−0.27 (*P* = 0.15)
Adjusted correlation	−**0.35 (** **P** = 0.02**)**	−0.22 (*P* = 0.12)	−**0.34 (** **P** = 0.04**)**	**0.52 (** **P** = 0.001**)**	0.21 (*P* = 0.23)	−0.08 (*P* = 0.58)	0.28 (*P* = 0.10)	−0.21 (*P* = 0.18)	−0.28 (*P* = 0.09)
Gender									
Unadjusted correlation	0.01 (*P* = 0.95)	−0.10 (*P* = 0.60)	−0.06 (*P* = 0.75)	−0.02 (*P* = 0.92)	0.08 (*P* = 0.69)	0.12 (*P* = 0.54)	0.01 (*P* = 0.95)	−0.08 (*P* = 0.66)	0.08 (*P* = 0.66)
Adjusted correlation	0.03 (*P* = 0.82)	0.06 (*P* = 0.65)	−0.01 (*P* = 0.93)	−0.15 (*P* = 0.30)	−0.11 (*P* = 0.51)	0.10 (*P* = 0.50)	−0.04 (*P* = 0.83)	−0.11 (*P* = 0.49)	0.03 (*P* = 0.88)
Total tear volume									
Unadjusted correlation	−0.25 (*P* = 0.19)	−0.26 (*P* = 0.16)	−0.12 (*P* = 0.53)	−0.36 (*P* = 0.05)	0.03 (*P* = 0.88)	**−0.60 (** **P** < 0.001**)**	**−0.52 (** **P** = 0.004**)**	−0.09 (*P* = 0.63)	−0.19 (*P* = 0.32)
Adjusted correlation	−0.17 (*P* = 0.25)	−0.22 (*P* = 0.12)	−0.08 (*P* = 0.64)	**−0.37 (** **P** = 0.02**)**	−0.09 (*P* = 0.61)	**−0.31 (** **P** = 0.04**)**	−0.32 (*P* = 0.06)	0.06 (*P* = 0.68)	−0.12 (*P* = 0.45)

**Table 3:** Medians (interquartile ranges) of released cytokine quantities (pg) at awakening (0 min) and 30 minutes after eye opening (∗ had *P* ≤ 0.007) in PKP patients and controls.

	IL-10		IL-13		IL-17	
	0 min	30 min	0 min	30 min	0 min	30 min
Controls	1.3 (1.5)	5.2∗ (4.6)	1.5 (1.0)	4.2∗ (2.3)	0.1 (0.4)	1.7∗ (2.2)
PKP patients	3.0 (3.7)	1.5 (3.0)	2.9 (2.2)	2.3 (1.7)	0.8 (1.1)	0.3 (1.1)

	IFN*γ*		CXCL8		CCL5	
	0 min	30 min	0 min	30 min	0 min	30 min

Controls	0.8 (0.8)	3.9∗ (3.7)	86.8 (52.5)	36.3∗ (52.6)	2.0 (1.1)	7.1∗ (6.0)
PKP patients	2.3 (2.2)	1.2 (2.1)	54.1 (54.6)	32.8∗ (34.8)	4.5 (5.3)	2.6 (4.2)
